# Projected Polar Bear Sea Ice Habitat in the Canadian Arctic Archipelago

**DOI:** 10.1371/journal.pone.0113746

**Published:** 2014-11-26

**Authors:** Stephen G. Hamilton, Laura Castro de la Guardia, Andrew E. Derocher, Vicki Sahanatien, Bruno Tremblay, David Huard

**Affiliations:** 1 Department of Biological Sciences, University of Alberta, Edmonton, AB, T6G 2E9 Canada; 2 Department of Earth and Atmospheric Sciences, University of Alberta, Edmonton, AB, T6G 2E9 Canada; 3 World Wildlife Fund Canada, PO Box 1750, Iqaluit, NU, X0A 0H0 Canada; 4 Atmospheric and Oceanic Sciences, McGill University, Room 823, Burnside Hall 805 Sherbrooke Street West, Montreal, QC H3A 0B9 Canada; 5 David Huard Solutions, Québec, G1W 4G8 Canada; Laval University, Canada

## Abstract

**Background:**

Sea ice across the Arctic is declining and altering physical characteristics of marine ecosystems. Polar bears (*Ursus maritimus*) have been identified as vulnerable to changes in sea ice conditions. We use sea ice projections for the Canadian Arctic Archipelago from 2006 – 2100 to gain insight into the conservation challenges for polar bears with respect to habitat loss using metrics developed from polar bear energetics modeling.

**Principal Findings:**

Shifts away from multiyear ice to annual ice cover throughout the region, as well as lengthening ice-free periods, may become critical for polar bears before the end of the 21^st^ century with projected warming. Each polar bear population in the Archipelago may undergo 2–5 months of ice-free conditions, where no such conditions exist presently. We identify spatially and temporally explicit ice-free periods that extend beyond what polar bears require for nutritional and reproductive demands.

**Conclusions/Significance:**

Under business-as-usual climate projections, polar bears may face starvation and reproductive failure across the entire Archipelago by the year 2100.

## Introduction

Observed changes in global climate have influenced Arctic sea ice cover more than most models have predicted [1], and ongoing sea ice declines indicate loss of maximum ice cover as well as older, thicker multiyear ice [2], [3]. These losses are modifying the Arctic marine ecosystems [4], [5], making Arctic and sub-Arctic marine mammals particularly vulnerable to climate change [6], [7]. Polar bears (*Ursus maritimus*) are inextricably linked to Arctic sea ice and are sensitive to sea ice loss [6]–[10]. Polar bears rely on sea ice as a platform for hunting, migrating, and mating, but are forced to move to land in regions where sea ice does not seasonally persist [11]–[14]. Energetics modeling and population projections indicate that continued sea ice loss with climate warming will negatively affect polar bear survival and reproduction potentially leading to population declines [15]–[18]. Moreover, of the ice that survives the melt season, insufficient snow cover may limit its viability as habitat for ringed seals (*Pusa hispida*), the primary prey species of polar bears [19].

**Figure 1:**
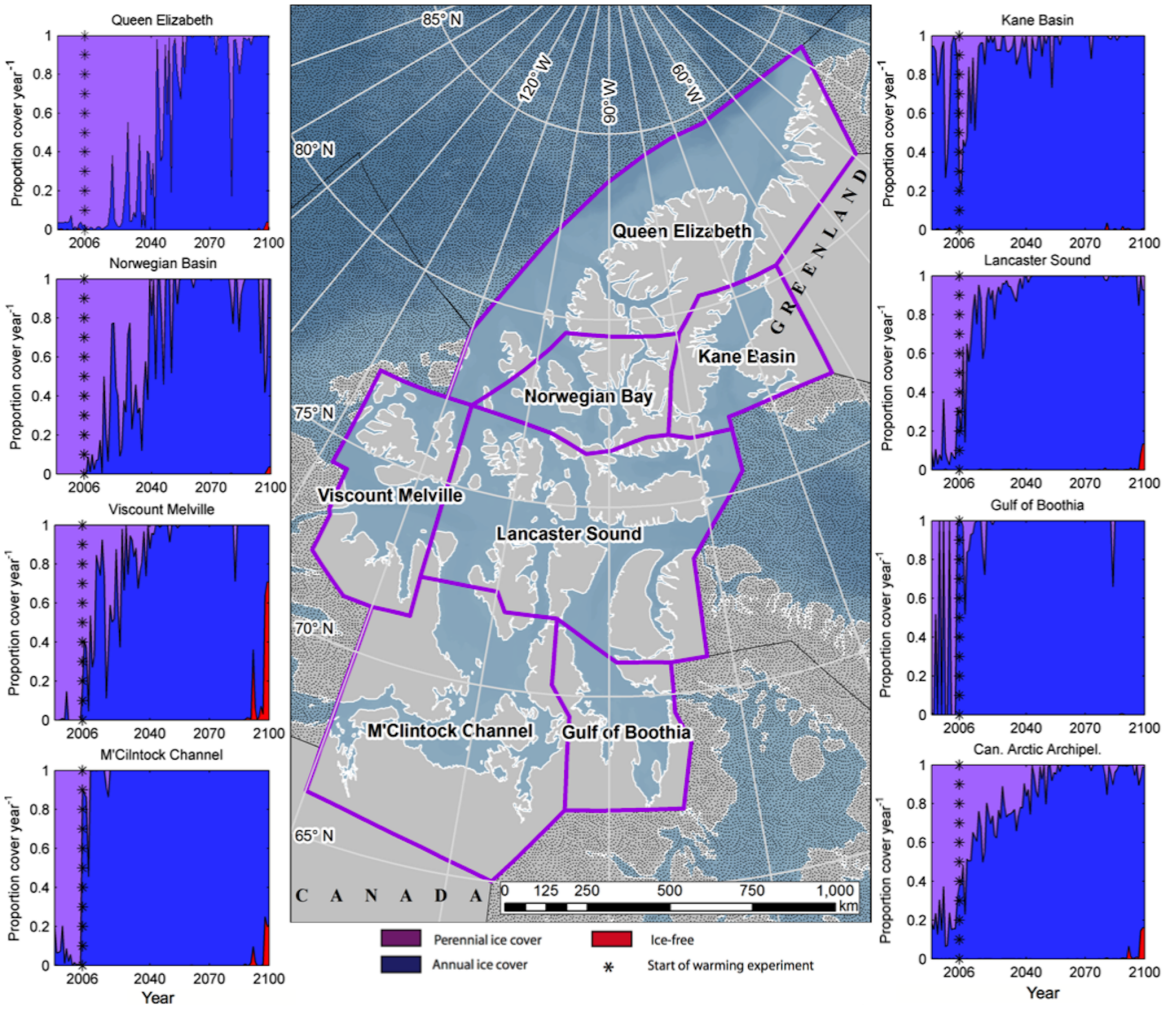
Projected dominance of seasonal sea ice in the polar bear populations of the Arctic Archipelago. The seven populations range from 65–85°N in latitude, with significant variation in the length of ice-free seasons. The proportion of multiyear ice, annual ice, and ice-free waters is given by regional means, and averaged over the total area.

Optimal polar bear habitat is predicted to decline in the 21^st^ century, with significant losses in the Hudson Bay and peripheral Arctic seas [15], [20], though greenhouse gas mitigation and geo-engineering strategies could limit some loss [21], [22]. Most sea ice modeling efforts have a crude representation of the geographically complex Canadian Arctic Archipelago (CAA) due to its many narrow channels, which are difficult to resolve. Nevertheless, 7 of the 19 recognized polar bear populations depend on the ice formed within or advected into the CAA (Figure 1). These 7 CAA populations comprise approximately one quarter of the estimated global polar bear population, while covering only 9.1% of the global polar bear range [23].

The CAA and Greenland were thought to have the greatest likelihood of sustaining polar bears to the end of the 21^st^ century [24] although based only on analysis of sea ice conditions in the very northern part of the CAA. Here we investigate the impact of projected warming on polar bears within the CAA from projected monthly mean sea ice concentration (SIC), ice thickness, and snow depth between 2006–2100 in comparison to previously established polar bear energetic needs. Polar bears are well-adapted to prolonged periods without food but lose body mass when fasting [24]–[28]. Body mass is already declining in some polar bear populations with negative consequences on survival and reproduction [16], [29], [30]. Energy budget models exist in which polar bear survival and reproductive rates can be tied to the availability of access to sea ice [17], [18]. Such models are based on the basic energy requirements of animals and are useful when predicting population changes under environmental conditions that have yet to be observed [31]. We examine the seasonality of sea ice and determine when the length of the ice-free period in the CAA may become critically limiting to polar bear foraging and thus negatively affect reproduction and survival.

## Materials and Methods

Global climate simulations contributed to the Coupled Model Intercomparison Project Phase 5 (CMIP5) are too coarse to effectively resolve the narrow channels of the CAA. Although numerically challenging, one solution is to dynamically downscale a global simulation onto a finer grid using a regional climate model. A comparison between over 30 different CMIP5 models led us to select one simulation from the Geophysical Fluid Dynamics Laboratory Coupled Physical Model (GFDL-CM3) driven by radiative scenario RCP8.5 to pilot the regional model (available from www.gfdl.noaa.gov/coupled-physical-model-cm3). This simulation includes a realistic spatial distribution of sea ice extent and thickness and simulates the trend in observed minimum sea ice extent during the observational record (1979–2013). This pilot simulation was dynamically downscaled using the ice-ocean Massachusetts Institute of Technology General Circulation Model (MITgcm) simulation in regional mode over the Arctic at a resolution of 18km (available from http://mitgcm.org). The 3-hourly atmospheric forcing fields from GFDL-CM3 were bias-corrected at the monthly scale using differences for variables (x,y,z) or ratios for variables (u,t,g) between the Japanese 25 year Reanalysis (JRA25) [32] and GFDL-CM3. These biases were calculated over the 2005–2011 period, arguably too short to compute climatological means, to smooth the transition from the JRA25 driven MITgcm simulation to the GFDL-CM3 driven simulation occurring at the start of 2012. We choose a period of 7 years to calculate the biases between the two forcing datasets because of the transitory nature of the climate in the early 21^st^ century with large trends in many of the Arctic climate forcing fields. MITgcm parameters were provided by Nguyen [33] and ocean boundary conditions taken from the Estimating the Circulation and Climate Change of the Ocean Phase 2 (ECCO2) experiment [34]. The MITgcm is run with time steps of 2-hours.

**Figure 2:**
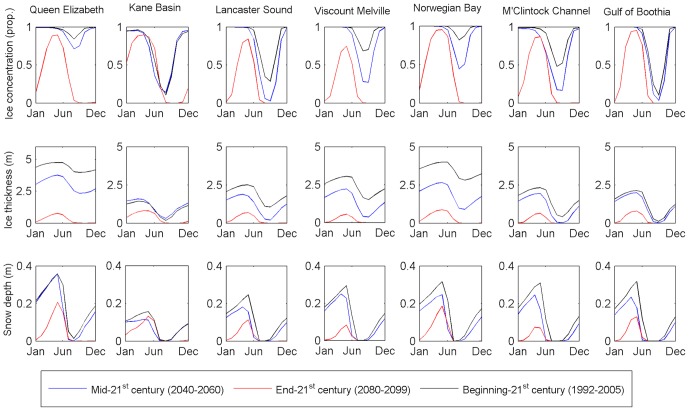
Changes in seasonal sea ice concentration (SIC), thickness, and snow depth over time by region. The mean ice-free season length (in months) for each time period is identifiable by segments of zero SIC or zero ice thickness. All values are monthly means over the respective time periods.

Our model projection is based on the RCP8.5 scenario, which estimates the global average radiative forcing at 8.5 W/m^2^ by 2100, and mean global temperature changes of ∼3.5°C in 2071–2100 when compared with the historical period of 1961–1990 [35], and represents a worst-case scenario. We compared the seasonal changes in the sea ice cycle between past (1992–2005), near future (2040–2050), and future (2080–2090) by comparing average SIC in each period by month (Figure 2). Population size, survival, and reproduction of polar bears have all been associated with the changes in the seasonal ice cycle, in particular with changes to the ice-free period [10], [29], [30]. We assume that effects on polar bears within the CAA will be comparable to those observed in other populations.

To study how habitat could change, we classified each pixel within the CAA as multiyear ice, annual ice, or ice-free. The classification was made based on the SIC of the pixel location over a given year [3]. Multiyear ice, which is ice that persists through the height of the melt season (typically March – September), is found when SIC ≥15% year-round. Should SIC dip to <15% before freeze-up begins, but be ≥15% at least once during the year prior, the pixel is classified as annual ice. Ice-free areas are defined as <15% SIC year-round. Polar bears typically avoid or abandon sea ice when concentrations drop below 30–50% although the rate of loss is also important [14], [29]. The cutoff of 15% we used is conservative because bears will occupy habitat with as little as 15% SIC [15], but higher concentrations are more closely associated with habitat use and successful predation [36], [37].

We defined a critical ice-free period as one in which sea ice was absent in sufficient concentration for ≥180 days or based on energy budget models [17], [18]. The ice-free period, with respect to polar bear habitat use, was assessed as the time between break-up (first month in a year with SIC<50%) until freeze-up begins (SIC≥10%). The values of 50% and 10% for break-up and freeze-up, respectively, are correlated with polar bear movements ashore and offshore in regions where there is a seasonal ice cycle [14], [29]. If all months had a mean SIC<10%, the ice-free season was twelve months. Conversely, if all months had SIC≥10% the ice-free season was zero months, which may be conservative regarding the impacts of low SIC on polar bears. For example, within the CAA polar bears select for habitat with 90% SIC year-round [38], and in pelagic Arctic regions polar bears tend towards SIC of 75–80% in spring, 65% in summer, 60% in fall, and 95% in winter [15], [39]. As with the SIC values, we assume that energetic restrictions on polar bears is consistent between populations.

## Results and Discussion

All of the CAA exhibits a shift from primarily multiyear ice cover to a primarily seasonally ice-free system by 2100, with the exception of Kane Basin and the Gulf of Boothia, which were largely annually ice-covered regions from the outset (Figure 1). In all cases, the final years of the simulation exhibit some proportion of year-round ice-free areas, where no such areas exist in most of the 21^st^ century.

While multiyear ice is not good hunting habitat due to its low prey abundance [38], [40], it provides an alternative habitat for polar bears who otherwise must move onto land during summer, and do not have to wait as long for the new ice to form in the autumn [41]. A shift towards annual ice may seem preferable because it is associated with greater hunting opportunities and ringed seals, the primary prey of polar bears [42], [43], may increase in abundance if multiyear ice is replaced by thinner annual ice [7]. However, sea ice must persist long enough for polar bears to take advantage of potential increases in prey density. With the exception of Kane Basin, all polar bear populations in the CAA reached 100% SIC between October and December in the late 20^th^ century, and a non-zero minimum SIC in August or September (Figure 2). By the late 21^st^ century, our simulation projects the southernmost regions (M'Clintock Channel, Gulf of Boothia) and central regions (Viscount Melville, Lancaster Sound) may be entirely ice-free for 5 months, and may no longer reach 100% SIC at maximum ice extent. In the north (Kane Basin, Norwegian Bay, Queen Elizabeth), the simulation estimates a 2–4 month ice-free season by the end of the 21^st^ century, and maximum concentrations <100% in 2080–2090. Ice thickness and snow depth exhibit similar declines throughout the CAA. Ice thickness in the late 20^th^ century was twice to nearly five times the thickness of the projected thickness in the same month of the late 21^st^ century.

Snow depth declines in part due to the reduction in sea ice surface and dates of formation, but also due to a predicted shift in precipitation from snow to rain [19]. Mean snow depth more than halves in the south and central CAA, with the most pronounced changes between the late 20^th^ and late 21^st^ centuries in the western regions (Viscount Melville, M'Clintock Channel). Furthermore, using a conservative estimate of a minimum 20 cm snow depth requirement for seal habitat [19], only the Queen Elizabeth and Norwegian Bay areas may be able to maintain significant ringed seal populations by the end of this century.

### Critical Ice-Free Periods

**Figure 3:**
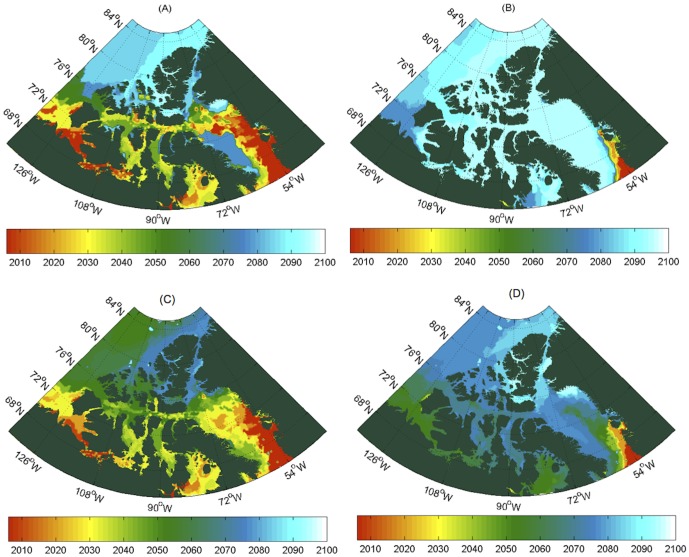
Critical ice-free periods for polar bear survival in the Canadian Arctic. The colors represent the year in which critical habitat loss is reached and never improves in subsequent years. Critical states are reached as starvation sets into adult males at (A) ≥120 days ice-free; (B) ≥180 days ice-free; and reproductive failure occurs in females with (C) break-up in July; and (D) break-up June.

Polar bears fare poorly when sea ice is absent for prolonged periods, losing body mass without the opportunity to hunt [26], [44]. Energetics modeling predicts that 2–3% of adult polar bear males could starve when the ice-free period reach 120 days and 9–21% could starve at 180 days of ice-free period with other age and sex classes even more vulnerable [17], [37], [45]. Similarly, early break-up of sea ice could result in reproductive failure in 55–100% of pregnant females [18]. The frequency of both events would have significant consequences for population trends in abundance. The thresholds established in these energetics models resulted in four types of critical ice-free periods, with the first two being relevant to male starvation rates, and the second two being relevant to female reproductive failure rates (Figure 3): (A) ice-free season >120 days; (B) ice-free season >180 days; (C) break-up occurs in July, and; (D) break-up occurs in June.

We find that sea ice conditions may become unsupportive of polar bear population persistence in the CAA and its surroundings by the late 21^st^ century with ice-free seasons reaching critical duration, and early break-ups occurring in parts of all populations we examined. Similarly, to the east of the CAA, the west coast of Greenland and much of Baffin Bay may no longer be suitable habitat for polar bears before 2050, though ice should persist along the east coast of Baffin Island until much later. Early break-up in the narrow channels of the central CAA may become critical in 2060–70s, whereas the adjacent coastlines of the open Arctic Ocean remain largely non-critical until near 2100.

**Figure 4:**
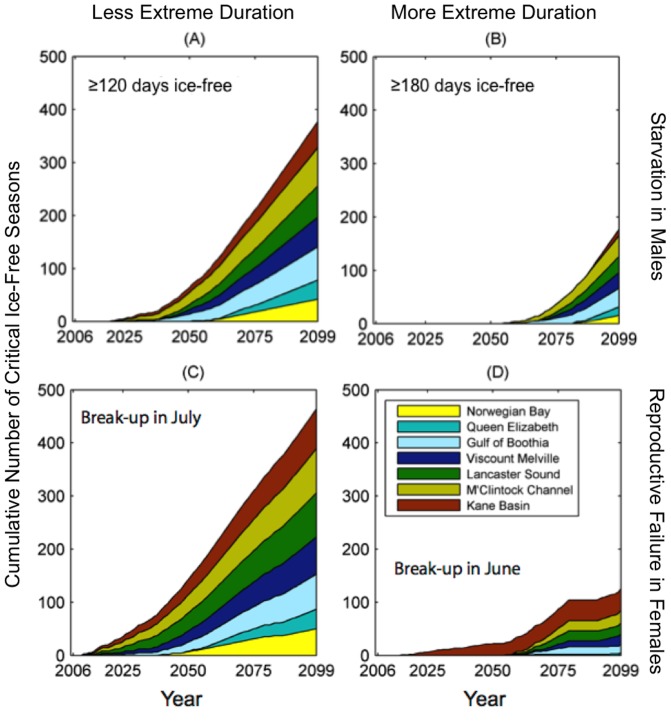
Cumulative number of critical ice-free seasons given by individual polar bear populations in the Canadian Arctic Archipelago. Each color represents the contribution of events in each population to the total number of critical seasons in a given year. Starvation in adult males occurs at (A) ≥120 days ice-free; (B) ≥180 days ice-free. Reproductive failure in females occurs when (C) break-up occurs in July; (D) break-up occurs in June.

It is important to consider that what we deem a critical point-of-no-return occurs once the ice-free period crosses our critical threshold and remains critical for the remainder of the modeled period. Nevertheless, it is feasible that single seasons, or clusters of seasons, may become critically ice-free before that point, with subsequent seasons being non-critical. As such, we examined the cumulative number of critical events for of the aforementioned critical periods (A–D) by population (Figure 4). We found that the less extreme critical durations (categories A and C) occur with lower frequency within the first decades of the simulation, and increase in frequency in later decades. When considering more extreme ice-free durations (categories B and D), critical events do not begin to occur until after 2050, with the exception of Kane Basin, which begins to experience break-up in June before 2020. Nevertheless, the frequency of critical events increases rapidly towards the end of the 21^st^ century.

### Implications for Conservation

Without exception, our simulation projects the sea ice habitat in all polar bear populations of the CAA may change from a multiyear to an annual ice system before the end of the century, and the remaining annual ice might not persist sufficiently long each year to allow hunting opportunities for polar bears as we currently understand them. Our model suggests that, by 2070, over 80% of the CAA might experience break-up in July, forcing pregnant females to retreat to land early, with possible negative effects on their reproductive output. Given that our study area comprises approximately one quarter of the world's polar bears, and nearly one-tenth of the total current habitat, our analyses project significant habitat loss and alteration under the business-as-usual model scenario used to estimate sea ice conditions over the coming 21^st^ century.

Conservation efforts to protect polar bear habitat in the Canadian Arctic should focus on regions that are slower to experience change in sea-ice concentration and ice-free period. The Queen Elizabeth and Norwegian Bay populations retain multiyear ice the longest, and their northerly fjords and channels consistently exhibit the fewest critical ice-free events. Nevertheless, by 2100 all regions of the study area may cross the critical point-of-no-return, putting the persistence of the CAA polar bear populations in jeopardy.

## References

[1] Stroeve JC, Kattsov V, Barrett A, Serreze M, Pavlova T, et al (2012) Trends in Arctic sea ice extent from CMIP5, CMIP3 and observations. Geophysical Research Letters 39.

[2] Maslanik JA, Fowler C, Stroeve J, Drobot S, Zwally J, et al (2007) A younger, thinner Arctic ice cover: Increased potential for rapid, extensive sea-ice loss. Geophysical Research Letters 34.

[3] Comiso JC (2012) Large Decadal Decline of the Arctic Multiyear Ice Cover. Journal of Climate 25: 1176–1193.

[4] Arrigo KR, van Dijken G, Pabi S (2008) Impact of a shrinking Arctic ice cover on marine primary production. Geophysical Research Letters 35.

[5] Bluhm BA, Gradinger R (2008) Regional variability in food availability for arctic marine mammals. Ecological Applications 18: S77–S96. 18494364 10.1890/06-0562.1

[6] Laidre KL, Stirling I, Lowry LF, Wiig Ø, Heide-Jorgensen MP, et al (2008) Quantifying the sensitivity of arctic marine mammals to climate-induced habitat change. Ecological Applications 18: S97–S125. 18494365 10.1890/06-0546.1

[7] Schipper J, Chanson JS, Chiozza F, Cox NA, Hoffmann M, et al (2008) The status of the world's land and marine mammals: Diversity, threat, and knowledge. Science 322: 225–230. 18845749 10.1126/science.1165115

[8] Stirling I, Derocher AE (1993) Possible impacts of climate warming on polar bears. Arctic 46: 240–245.

[9] Derocher AE, Lunn NJ, Stirling I (2004) Polar bears in a warming climate. Integrative and Comparative Biology 44: 163–176. 21680496 10.1093/icb/44.2.163

[10] Rode KD, Amstrup SC, Regehr EV (2010) Reduced body size and cub recruitment in polar bears associated with sea ice decline. Ecological Applications 20: 768–782. 20437962 10.1890/08-1036.1

[11] Stirling I (1974) Midsummer observations on behavior of wild polar bears (*Ursus maritimus*). Canadian Journal of Zoology-Revue Canadienne De Zoologie 52: 1191–1198.

[12] Ramsay MA, Stirling I (1986) On the mating system of polar bears. Canadian Journal of Zoology-Revue Canadienne De Zoologie 64: 2142–2151.

[13] Schliebe S, Rode KD, Gleason JS, Wilder J, Proffitt K, et al (2008) Effects of sea ice extent and food availability on spatial and temporal distribution of polar bears during the fall open-water period in the Southern Beaufort Sea. Polar Biology 31: 999–1010.

[14] Cherry SG, Derocher AE, Thiemann GW, Lunn NJ (2013) Migration phenology and seasonal fidelity of an Arctic marine predator in relation to sea ice dynamics. Journal of Animal Ecology 82: 912–921. 23510081 10.1111/1365-2656.12050

[15] Durner GM, Douglas DC, Nielson RM, Amstrup SC, McDonald TL, et al (2009) Predicting 21st-century polar bear habitat distribution from global climate models. Ecological Monographs 79: 25–58.

[16] Hunter CM, Caswell H, Runge MC, Regehr EV, Amstrup SC, et al (2010) Climate change threatens polar bear populations: a stochastic demographic analysis. Ecology 91: 2883–2897. 21058549 10.1890/09-1641.1

[17] Molnár PK, Derocher AE, Thiemann GW, Lewis MA (2010) Predicting survival, reproduction and abundance of polar bears under climate change. Biological Conservation 143: 1612–1622.

[18] Molnár PK, Derocher AE, Klanjscek T, Lewis MA (2011) Predicting climate change impacts on polar bear litter size. Nature Communications 2. 10.1038/ncomms1183 PMC3105343 21304515

[19] Hezel PJ, Zhang X, Bitz CM, Kelly BP, Massonnet F (2012) Projected decline in spring snow depth on Arctic sea ice caused by progressively later autumn open ocean freeze-up this century. Geophysical Research Letters 39.

[20] de la Guardia LC, Derocher AE, Myers PG, van Scheltinga ADT, Lunn NJ (2013) Future sea ice conditions in Western Hudson Bay and consequences for polar bears in the 21st century. Global Change Biology 19: 2675–2687. 23716301 10.1111/gcb.12272

[21] Amstrup SC, DeWeaver ET, Douglas DC, Marcot BG, Durner GM, et al (2010) Greenhouse gas mitigation can reduce sea-ice loss and increase polar bear persistence. Nature 468: 955–958. 21164484 10.1038/nature09653

[22] Tilmes S, Jahn A, Kay JE, Holland M, Lamarque J-F (2014) Can regional climate engineering save the summer Arctic sea ice? Geophysical Research Letters 41: 880–885.

[23] IUCN/PBSG (2013) Status table for the world's polar bear subpopulations. International Polar Bear Specialist Group, editor. Available online at http://pbsg.npolar.no/en/.

[24] Amstrup SC, Marcot BG, Douglas Dc (2008) A Bayesian Network Modeling Approach to Forecasting the 21st Century Worldwide Status of Polar Bears. In: DeWeaver ET, Bitz CM, Tremblay LB, editors. Arctic Sea Ice Decline: Observations, Projections, Mechanisms, And Implications. Washington: Amer Geophysical Union. 213–268.

[25] Watts PD, Hansen SE (1987) Cyclic starvation as a reproductive strategy in the polar bear. Symposia of the Zoological Society of London 57: 305–318.

[26] Ramsay MA, Stirling I (1988) Reproductive-biology and ecology of female polar bears (*Ursus maritimus*). Journal of Zoology 214: 601–634.

[27] Derocher AE, Stirling I (1995) Temporal variation in reproduction and body-mass of polar bears in Western Hudson-Bay. Canadian Journal of Zoology 73: 1657–1665.

[28] Atkinson SN, Ramsay MA (1995) The effects of prolonged fasting of the body-composition and reproductive success of female polar bears (*Ursus maritimus*). Functional Ecology 9: 559–567.

[29] Robbins CT, Lopez-Alfaro C, Rode KD, Toien O, Nelson OL (2012) Hibernation and seasonal fasting in bears: the energetic costs and consequences for polar bears. Journal of Mammalogy 93: 1493–1503.

[30] Stirling I, Lunn NJ, Iacozza J (1999) Long-term trends in the population ecology of polar bears in western Hudson Bay in relation to climatic change. Arctic 52: 294–306.

[31] Regehr EV, Lunn NJ, Amstrup SC, Stirling L (2007) Effects of earlier sea ice breakup on survival and population size of polar bears in western Hudson bay. Journal of Wildlife Management 71: 2673–2683.

[32] Kooijman SALM (2010) Dynamic energy budget theory for metabolic organisation. Cambridge UK: Cambridge University Press.

[33] Onogi K, Tslttsui J, Koide H, Sakamoto M, Kobayashi S, et al (2007) The JRA-25 reanalysis. Journal of the Meteorological Society of Japan 85: 369–432.

[34] Nguyen AT, Menemenlis D, Kwok R (2011) Arctic ice-ocean simulation with optimized model parameters: Approach and assessment. Journal of Geophysical Research-Oceans 116.

[35] Menemenlis D, Campin J, Heimbach P, Hill C, Lee T, et al.. (2008) ECCO2: High resolution global ocean and sea ice data synthesis. 42 p.

[36] Christensen OB, Goodess CM, Harris I, Watkiss P (2011) European and Global Climate Change Projections: Discussion of Climate Change Model Outputs, Scenarios and Uncertainty in the EC RTD ClimateCost Project; Watkiss P, editor. Stockholm, Sweden: Stockholm Environment Institute. 28 p.

[37] Rode KD, Regehr EV, Douglas DC, Durner G, Derocher AE, et al (2013) Variation in the response of an Arctic top predator experiencing habitat loss: feeding and reproductive ecology of two polar bear populations. Global Change Biology 20: 76–88. 23913506 10.1111/gcb.12339

[38] Pilfold NW, Derocher AE, Richardson E (2014) Influence of intraspecific competition on the distribution of a wide-ranging, non-territorial carnivore. Global Ecology and Biogeography 23: 425–435.

[39] Ferguson SH, Taylor MK, Messier F (2000) Influence of sea ice dynamics on habitat selection by polar bears. Ecology 81: 761–772.

[40] Arthur SM, Manly BFJ, McDonald LL, Garner GW (1996) Assessing habitat selection when availability changes. Ecology 77: 215–227.

[41] Kingsley MCS, Stirling I, Calvert W (1985) The distribution and abundance of seals in the Canadian High Arctic, 1980–82. Canadian Journal of Fisheries and Aquatic Sciences 42: 1189–1210.

[42] Amstrup SC, Durner GM, Stirling I, Lunn NN, Messier F (2000) Movements and distribution of polar bears in the Beaufort Sea. Canadian Journal of Zoology-Revue Canadienne De Zoologie 78: 948–966.

[43] Smith TG (1980) Polar bear predation of ringed and bearded seals in the land-fast sea ice habitat. Canadian Journal of Zoology-Revue Canadienne De Zoologie 58: 2201–2209.

[44] Thiemann GW, Iverson SJ, Stirling I (2008) Polar bear diets and Arctic marine food webs: insights from fatty acid analysis. Ecological Monographs 78: 591–613.

[45] Polischuk SC, Norstrom RJ, Ramsay MA (2002) Body burdens and tissue concentrations of organochlorines in polar bears (*Ursus maritimus*) vary during seasonal fasts. Environmental Pollution 118: 29–39. 11996380 10.1016/s0269-7491(01)00278-0

[46] Molnár PK, Derocher AE, Thiemann GW, Lewis MA (2014) Corrigendum to “Predicting survival, reproduction and abundance of polar bears under climate change” [Biol. Conserv. 143 (2010) 1612–1622]. Biological Conservation 177: 230–231.

